# Whole genome sequencing and antibiotic diffusion assays, provide new insight on drug resistance in the genus *Pedobacter*

**DOI:** 10.1093/femsec/fiaa088

**Published:** 2020-05-09

**Authors:** Ingvild Falkum Ullmann, Anders Benteson Nygaard, Hege Smith Tunsjø, Colin Charnock

**Affiliations:** 1 Oslo Metropolitan University, Faculty of Health Sciences, Department of Life Sciences and Health, NO-0130, Oslo, Norway; 2 Department of Microbiology and Infection control, Akershus University Hospital, Lørenskog, Norway

**Keywords:** antimicrobial resistance, minION sequencing, *Pedobacter spp*, environmental microbiology, drinking water

## Abstract

A total of four strains of the ‘environmental superbug’ *Pedobacter* isolated from sludge produced at Norwegian drinking water treatment plants, were characterized by whole genome sequencing and antibiotic susceptibility assays. As with previous studies on members of this genus, we found that the isolates were multi-drug resistant, and that this resistance included clinically important beta-lactams, aminoglycosides and the fluoroquinolone ciprofloxacin. Using the minION sequencing platform (Oxford Nanopore Technologies) combined with HiSeq PE150 Illumina sequencing data, the four isolates were assembled into genomes of single contigs. Analysis of the genomes revealed potential genetic factors possibly underlying some of the specific resistances observed. Metallo-beta-lactamase activity was detected in one isolate, and the same isolate contained a putative metallo-betalactamase gene resembling *pedo-2*. Furthermore, several genes related to multidrug efflux systems were found using the resistance database CARD. Additionally, the present study extends our knowledge on the phylogeny of this genus, adding four new genomes to the existing 50.

## Teaser

Whole genome sequencing of the environmental superbugs™ *Pedobacter spp*. provides insights on their multi-drug resistance.

## INTRODUCTION

Antibiotic resistance accounts for many thousands of deaths annually, and its projected increase has led to its recognition by the World Health Organization (WHO) as a major global health threat (WHO 2014). However, there are still many unanswered questions concerning the different processes that lead to the development, mobilization (e.g. appearance on plasmids or transposons) and the spread of resistance genes. Studies on antibiotic resistance have been mainly in the domain of clinicians. Over recent years, however, the role of the environment as an important source of and dissemination route for resistance has been increasingly recognized (Walsh and Duffy 2013; Narciso-da-Rocha and Manaia 2016; Bengtsson-Palme, Kristiansson and Larsson 2018). Several authors have highlighted the need to take a holistic perspective on antibiotic resistance, including humans, animals and the external environment—a so-called one-health approach (Finley *et al*. 2013).

Sludge production at drinking water plants is the first step of the purification process. The material is easily obtainable and because it derives from large areas (lakes and the surrounding catchment), it is well suited to the study of the presence of antibiotic resistant bacteria and resistance genes in the environment. Furthermore, resistant bacteria and genes found in drinking water sludge have a short route to the human microbiome, either directly through drinking water, or by the use of sludge as a fertilizer in agriculture. Antibiotic resistance genes (ARG) may represent a high fitness cost for bacteria. In the absence of a selection pressure, these are more likely to be lost, particularly if located on mobile genetic elements (Bengtsson-Palme, Kristiansson and Larsson 2018). Determinants of resistance to metals and biocides in the environment, have been found to be co-localized with antibiotic resistance genes on mobile genetic elements (Baker-Austin *et al*. 2006; Seiler and Berendonk 2012; Wales and Davies 2015; Pal *et al*. 2017). Such determinants have the potential of inducing a selective pressure for the development of antibiotic resistance, even in the absence of antibiotics (Pal *et al*. 2015). Sludge is a highly-concentrated material arising from suspended particles in the drinking water source and solutes, which can include metal-based coagulants used in the clarification process. This may create a selective pressure favoring a co-selection of antibiotic resistance and thus the maintenance of resistance genes.

In a recent study of aminoglycoside resistance among bacteria isolated from drinking water sludge in Norway, we found that about 24% of the resistance resided in members of the Gram-negative genus *Pedobacter* (Ullmann *et al*. 2019). This suggests that members of the genus have a physiology well suited to survival and proliferation in this environmental niche. *Pedobacter* spp. have been found in a variety of other environments, including soil, fresh-water, glaciers, mines and fish (Gallego, Garcia and Ventosa 2006; Ana Teresa Wei *et al*. 2018; Viana *et al*. 2018b). The genus *Pedobacter* proposed by Steyn *et al*. (1998), belongs to the family *Sphingobacteriaceae* in the phylum Bacteroidetes. There are now 91 recognized species of *Pedobacter* (NCBI: txid84567 Accessed 18th of April 2020). Although there is some variation in resistance profiles, a survey of the descriptions of *Pedobacter* species in the literature, shows resistance against clinically important antibiotics to be quite typical. Indeed, *Pedobacter* has recently been described as an *environmental superbug* with multiple antibiotic resistance mechanisms (Viana *et al*. 2018a). Although there are 50 genome assemblies of *Pedobacter* species available, there have been few attempts to identify the resistance determinants involved. Multidrug resistant (MDR) environmental bacteria such as *Pedobacter*, might provide clues on how ARG cross into and establish themselves in the clinical setting. Two recent studies (Gudeta *et al*. 2016a; Viana *et al*. 2018b) report the successful cloning and expression of *Pedobacter* Metallo-beta-lactamase (MBL) in *Escherichia coli*, further underlining the potential significance of these enzymes should they appear in strains of clinical relevance.

We hypothesize that whole genome sequencing (WGS) might shed light on multiresistance in *Pedobacter* and the possibility of the transfer of ARG to more clinically-relevant species. In the present study, four MDR sludge isolates were selected for detailed phenotypic resistance characterization and whole genome sequencing analysis by HiSeq150 (Illumina Inc.) and minION (Oxford Nanopore Technologies) platforms.

## MATERIALS AND METHODS

### Isolates

A total of 22 strains of previously identified *Pedobacter* isolates (Ullmann *et al*. 2019) were initially tested for susceptibility to a panel of antibiotics as described below (Supplementary material). A total of four of these isolates, were then chosen for whole genome sequencing and an extended susceptibility assay, including the determination of MIC-values. The criteria used to select the isolates were: differences in colony morphology and/or color, their ability to grow well on Mueller Hinton agar II for susceptibility testing, and on the results of the limited susceptibility testing for all 22 strains (supplementary material). Furthermore, the four isolates were also selected based on their origin at different WTPs. Information on the isolates is presented in Table 1.

**Figure 1.**
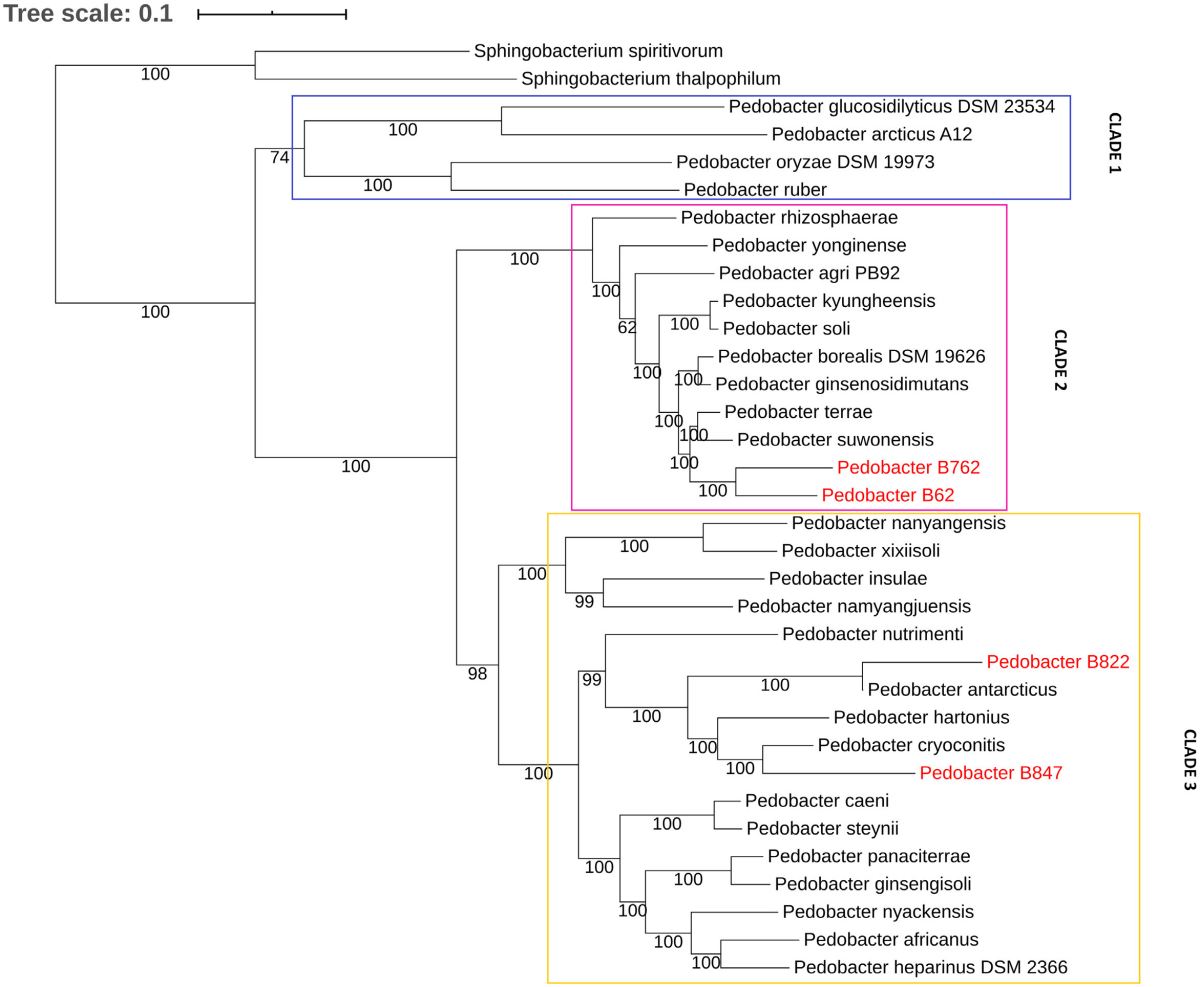
Phylogenetic tree of *Pedobacter spp*. Using the online tool PATRIC, the phylogeny of 32 different *Pedobacter* genomes was produced with RAxML and Jackknife (50%). One minor clade and two major clades are indicated. The four isolates investigated in this study spread out on two different clades and are given in red. Isolates B7_64 and B6_2 have a similar phylogeny and group together in the proposed Clade 2. Isolate B8_22 is closely related to *Pedobacter antarcticus*, while isolate B8_47 branches with *Pedobacter cryoconitis*. The tree was rooted using two strains of *Sphingobacterium*.

**Table 1.** Summary information on the four isolates chosen for further investigation.

Isolate*	Municipality	County	WTP	Color
1–B6_2	Askim	Østfold	WTP16	Pink
2–B7_62	Lillestrøm	Akershus	WTP18	Pink
3–B8_22	Froland	Aust-Agder	WTP20	Yellow
4–B8_47	Enebakk	Akershus	WTP21	White

* Numbering scheme adapted from previous study (reference)

### Disc diffusion assay for antibiotic sensitivity

A total of 22 *Pedobacter* isolates were initially tested for phenotypic susceptibility to the following antibiotics: amikacin, gentamycin, ciprofloxacin, imipenem and sulfamethoxazole/trimethoprim STX (Supplementary data). The four isolates selected for further analyses (Table 1) were additionally tested for sensitivity to aztreonam, ampicillin, cefotaxime, nitrofurantoin, ticarcilin, tigecycline, trimethoprim, tobramycin and cefoxitin. Antibiotic concentrations were as described in Table 2. Tests were based on the EUCAST disc diffusion method (EUCAST 2018). However, because standardized test conditions and breakpoints are not available for *Pedobacter*, some modifications were made. In brief, isolates were grown for 48 h at 28°C on cation-adjusted Mueller Hinton agar II (MHA) (Oxoid, Basingstoke, UK) before a small number of colonies were suspended in 0.9% sterile NaCl to a concentration corresponding to MacFarland 0.5 measured spectrophotometrically. Suspensions were swabbed onto MHA (14 cm plates), and after addition of antibiotic discs plates were incubated for 36–48 h at 28°C before zones of inhibition were measured. *E. coli* ATCC 25922, for which EUCAST breakpoints are available, was used as quality control. Zone diameters were read and interpreted using the EUCAST 8.1 document (EUCAST 2018). Susceptibilities were recorded as S, I or R based on breakpoints for *E. coli*. In instances where complete inhibition (no zones) were obtained, this observation was recorded alongside the susceptibility category.

**Table 2.** Extended phenotype susceptibility test of four selected *Pedobacter* isolates.

Antibiotic	Drug group	Disc content, µg	B6_2	B7_62	B8_22	B8_47
Amikacin	Aminoglycoside	30	R	R	R	R
Gentamycin	Aminoglycoside	10	R	R	R	I
Tobramycin	Aminoglycoside	10	R	R	R	R
Trimethoprim	Pyrimidine	5	S	S	S	S
Ampicillin	Beta-lactam (penicillin)	10	R	R	R	R
Aztreonam	Beta-lactam (monobactam)	30	R	R	R	R
Cefotaxime	Beta-lactam (Cephalosporin)	5	R	R	R	R
Cefoxitin	Beta-lactam (Cephalosporin)	30	R	R	R	R
Imipenem	Beta-lactam (Carbapenem)	10	R	S	S	I
Ticarcillin	Beta-lactam (Penicillin)	75	R	R	R	R
Ciprofloxacin	Fluoroquinolones	5	R	R	R	S
Nitrofurantoin	Nitrofuran derivatives	100	S	S	S	S
Tigecycline	Tetracycline	15	S	S	S	S
Sulfamethoxazole /Trimethoprim (SXT)	Sulfonamide/ pyrimidine	25	S	S	S	S

R = resistant, S = sensitive, I = intermediate resistance. *E. coli* ATCC 25922 was used as quality control. Zone diameters were read and interpreted using the EUCAST 8.1 document (EUCAST 2018).

#### Determination of MIC-values (broth microdilution)

The MIC values of 15 antibiotics for the 4 strains chosen for in-depth characterization were determined using the broth microdilution method, with Sensititre GN4F Gram-negative minimum inhibitory concentration (MIC) plate panels (ThermoFisher Scientific, Waltham, MA) The assay was performed essentially as described in the manufacturers’ protocol. In brief a 0.5 McFarland suspension of each isolate and the control strain *E. coli* ATCC 25922 diluted in cation adjusted MH broth, were added to the wells of the plate. The plates were incubated at 28°C for 48 h, and inspected regularly between 18–48 h using a concave mirror reader for any sign of growth. The MIC was defined as the lowest concentration of an antimicrobial agent that completely inhibited the growth after 48 h incubation. Experiments were repeated with new cultures.

### Detection of carbapenemase activity

Three different test methods were used to detect carbapenemase activity in the four *Pedobacter* isolates chosen for further study.

The Carbapenem Inactivation Method (CIM) for detection of carbapenemase activity was done and interpreted essentially as previously described (van der Zwaluw *et al*. 2015). In brief, *Pedobacter* isolates were grown on MHA for 36 h at 28°C. Thereafter, a full 10 µL inoculation loop of each *Pedobacter* strain and *E. coli* ATCC 25922 was suspended separately in 400 µL sterile distilled water. Subsequently a disc containing 10 µg imipenem (Oxoid) was immersed in the suspension and incubated for two hours at 28°C. Additional tubes contained an antibiotic disc and 10 µL 100 mM EDTA to inhibit the activity of metallo-beta-lactamases (Pierce *et al*. 2017). After incubation, discs were removed aseptically, and excess liquid was removed by placing the disc on sterile filter paper prior to transfer to a MHA plate seeded with a MacFarland 0.5 suspension of *E. coli* ATCC 25922. The diameter of zones of inhibition were read after incubation for 18–24 h at 35°C. Antibiotic discs not previously placed in contact with bacteria were also included on the plate.

The Modified Hodge Test (MHT) was performed as recommended by CLSI (CLSI 2012). MHA plates were seeded with *Klebsiella pneumoniae* CCUG 45421 (indicator strain). One *Pedobacter* isolate per plate was inoculated onto each plate in three straight lines from the centre to the edge of the plate, before discs containing respectively, ertamepenem 10 µg, meropenem 10 µg and imipenem 10 µg were placed onto the agar. The plates were incubated at 28°C for 20 hours.

To test for carbapeneamse activity in the four *Pedobacter* isolates, the qualitative colorimetric β CARBA test (Bio-Rad, CA) was performed according to the manufacturer's protocol.

The MBL producing *Pseudomonas aeruginosa* CCUG 59347 was used as positive control and *K. pneumoniae* CCUG 45421 was used as negative control in all experiments.

### Further characterization of carbapenemase activity

To further characterize the carbapenemase activity of strain B6_2 two different tests were performed:

The KPC/Metallo-ß-lactamase confirm kit (Rosco, Taastrup, DK) was performed according to the supplied protocol. The plates were incubated at 28°C until growth was visible, which was after 48 hours for the *Pedobacter* isolate. The diameters of inhibition zones were measured. To ensure accurate recording of the inhibition zones, two tablets (meropenem 10 µg and meropenem + inhibitor) were added to each plate.

The MIC Test Strip Synergy Applicator System (MTS-SAS) from Liofilchem® (Integrated Sciences, NSW, AU) was used to test for MBL activity. MIC strips with Imipenem 4–256 μg/mL in one end and Imipenem + EDTA 1–64 μg/mL in the other end were placed on MHA plates seeded with MacFarland 0.5 suspensions of the *Pedobacter* isolates. The plates were incubated at 28°C and MIC results were read at 24 and 48 h.

### DNA extraction

DNA was extracted in parallels of three using the PureLink Genomic DNA Mini Kit (Invitrogen, Carlsbad, CA). Extractions were performed as described in the manufacturer's protocol for Gram-negative bacteria. The incubation time of the lysis step was set to three hours. DNA concentrations were measured using Qubit Fluorometric quantification (ThermoFisher Scientific, MA) employing the dsDNA Broad Range Assay as described by the Manufacturer.

### Whole genome sequencing

Whole genome sequencing using the minION platform was performed using The Rapid Barcoding Sequencing Kit (SQK-RBK004) (Oxford Nanopore Technologies, Oxford, GB), according to the manufacturer's protocol and starting with 400 ng DNA from each isolate. Prior to library preparation, the optional clean-up protocol with AMPure XP beads (Beckman Coulter, CA) was performed to increase throughput by concentrating the pooled barcoded material. The final library-prep was loaded onto a R9.4 MinION Flow Cell (FLO-MIN107). The sequencing run was performed on a minION MK1b (Oxford Nanopore Technologies) device in real-time through the minKNOW platform using the MIN107_SQK-RBK004 protocol. The run time was 48 h. DNA was additionally sent for library preparation and Illumina sequencing by Novogene Bioinformatics Technology Co., Ltd (Beijing, China). Library preparation and sequencing was performed on the Illumina HiSeq PE150 platform.

### Processing of sequencing data

Basecalling and data conversion of minION sequencing data were performed using Albacore version 1.2.4 (https://github.com/Albacore/albacore). After basecalling, adapters were trimmed from the sequence reads using PoreChop version 0.2.3 (https://github.com/rrwick/Porechop). De-novo assembly was performed applying Canu version 1.6 (Koren *et al*. 2017). *De Novo* assembly of Illumina reads was done using the SPAdes pair-end library pipeline (https://github.com/ablab/spades) (Nurk *et al*. 2013). Quality reports for both Illumina and minION assembled genomes were generated using QUAST (Gurevich *et al*. 2013). Hybrid assemblies merging minION and Illumina data into single genome assemblies for each isolate were performed by Novogene Bioinformatics Technology Co., Ltd (Beijing, China) using the SOAPdenovo (https://github.com/aquaskyline/SOAPdenovo2) pipeline (Luo *et al*. 2012). All sequencing data can be found publicly available at DOI 10.6084/m9.figshare.7637657.

### Phylogeny based on whole genome sequencing

The Phylogenetic Tree Building Service, an online tool provided by PATRIC (3.5.27) (Wattam *et al*. 2017), was used to create a phylogenetic tree of 32 different *Pedobacter* spp. genomes using the Maximum likelihood (RAxML) method (Stamatakis 2014). In addition to the four genomes produced in this study, 28 genomes available through PATRIC were included. The tree was rooted using two strains of *Sphingobacterium*.

### Resistome data analysis

Genes conferring drug resistance were identified using the nucleotide input of the RGI (Resistance Gene Identifier) tool in loose mode against the Comprehensive Antibiotic Resistance Database (CARD) (McArthur *et al*. 2013; McArthur and Wright 2015; Jia *et al*. 2017). The open source software R, (Team 2017) was used to create visual representations of these findings. The sequence alignment tool Jalview v. 2.10.5 (Waterhouse *et al*. 2009) was used to align sequence from isolates with references retrieved from GenBank. The available online tool ICEfinder (Liu *et al*. 2018) (http://202.120.12.136:7913/ICEfinder/ICEfinder.html) was used to search for potential mobile genetic elements present in the flanking regions of the ARG detected by RGI.

## RESULTS

### Antibiotic susceptibility

#### Disc diffusion assay

A total of 22 *Pedobacter* isolates were initially tested for susceptibility using a small panel of antibiotics (Supplementary material). Results confirmed that members of this genus are generally MDR. Four isolates were chosen for susceptibility testing to in total 14 different antibiotics, representing seven different major drug groups (Table 2). Testing showed that the isolates have similar phenotypic resistance patterns. A total of four (trimethoprim, nitrofurantoin, tigecycline and SXT) out of 14 of the tested antibiotics gave complete inhibition of bacterial growth for all four isolates, while seven (amikacin, tobramycin, ampicillin, aztreonam, cefotaxime, cefoxitin and ticarcillin) of the drugs did not have any effect on growth. The three remaining drugs (imipenem, gentamicin and ciprofloxacin) had variable effects on the four isolates. Imipenem, one of six beta-lactam antibiotics tested in this assay, gave different patterns of susceptibility for the four bacterial isolates. The growth of isolates B8_22 and B7_62 was inhibited completely, while B8_47 showed an intermediate resistant phenotype. Only isolate B6_2 was fully resistant to imipenem. The growth of isolate B8_47 was completely inhibited by the fluoroquinolone ciprofloxacin, while the other isolates were resistant towards this antibiotic. The results for the quality control strain Escherichia coli ATCC 25922 were within the acceptable inhibition zone ranges listed for this strain in EUCAST QC Tables v. 10.0, valid from 2020–01-01, for all antibiotics tested.

#### Broth microdilution assay

The MIC values of the four *Pedobacter* isolates and the control strain *E. coli* ATCC 25922 were determined for 15 different antibiotics. MIC values obtained for the control strain *E. coli* ATCC 25922 were those given in the EUCAST document for all antibiotics tested. As 36–48 h incubation was required to given sufficient growth of all *Pedobacter* isolates tested, MIC values for the *E. coli* control were read after 48 h as well as 24 h to confirm antibiotic stability. The MIC values of the control were unchanged after additional incubation. The MIC-values (Table 3) for *Pedobacter* spp. after 48 h incubation, were in general agreement with the results of the disc diffusion assay (Table 2). Non-alignment was only seen with respect to the sensitivity of B8_47 to ciprofloxacin. Based on the disc diffusion assay this isolate showed complete sensitivity to ciprofloxacin, whereas an MIC value > 2 µg/mL, indicating a resistant phenotype, was obtained in the broth microdilution assay. The MIC assay results indicate that B6_2 is resistant to the included carbapenems (doripenem, ertapenem, imipenem, meropenem). Of the four carbapenems, only ertapenem showed a similar resistance pattern between the four *Pedobacter* isolates (Table 3). B8_47 showed some resistance to doripenem, imipenem and meropenem. However, the MIC values were lower than those of B6_2.

**Table 3.** MIC values determination for 15 antibiotics and the corresponding phenotype susceptibility test results.

	B6_2		B7_62		B8_22		B8_47		E. Coli ATTC 25922
	MIC	Disc diffusion test	MIC	Disc diffusion test	MIC	Disc diffusion test	MIC	Disc diffusion test	MIC
Amikacin	8	R	> 32	R	> 32	R	> 32	R	< 8
Gentamycin	> 8	R	> 8	R	> 8	R	> 8	I	< 2
Tobramycin	> 8	R	> 8	R	> 8	R	> 8	R	< 2
Ampicillin	8	R	> 16	R	> 16	R	> 16	R	< 8
Aztreonam	> 16	R	> 16	R	> 16	R	> 16	R	< 1
Cefazolin	> 16	NA	> 16	NA	> 16	NA	> 16	NA	1
Piperacillin	16	NA	32	NA	> 64	NA	> 64	NA	< 16
Imipenem	4	R	< 0.5	S	< 0.5	S	2	I	< 0.5
Meropenem	> 8	NA	< 0.5	NA	0.5	NA	2	NA	< 0.5
Doripenem	> 4	NA	< 0.5	NA	< 0.5	NA	1	NA	< 0.5
Ertapenem	4	NA	4	NA	2	NA	4	NA	<0.25
Ciprofloxacin	> 2	R	> 2	R	> 2	R	> 2	S	< 0.5
Tetracycline	< 4	NA	< 4	NA	< 4	NA	< 4	NA	< 4
Tigecycline	< 1	S	< 1	S	< 1	S	1	S	< 1
SXT	<2/38	S	<2/38	S	<2/38	S	<2/38	S	<2/38

NA = Not Applicable, the respective antibiotic was not included in the phenotypic susceptibility test (Table 2).

#### Carbapenemase activity

Enhanced growth of the indicator strain in the Modified Hodge Test was only obtained through contact with the positive control. Neither the *Pedobacter* isolates or the negative control affected the growth of the indicator strain.

The results of the CIM test showed that zones of inhibition obtained using discs of imipenem were in the expected range for *E. coli* ATCC 25922 (EUCAST 2018), and indicated complete susceptibility of the strain. Discs incubated in the presence of *Pedobacter* or *E. coli* prior to susceptibility testing, showed in all instances a reduction in zone sizes of about 5–10 mm relative to non-incubated discs. However, there was no indication that this was caused by factors other than loss of antibiotic from the discs into the bacterial suspension during incubation: suspensions of all strains, including the non-carbapenemase producing *E. coli* 25922, caused approximately equal reductions in zone sizes and no effect of EDTA (inhibitor of metal-beta-lactamases) was observed in any instance.

The results of the ß-Carba test (Bio-Rad), shown in Table 4, indicate that B6_2 is positive for carbapenemase activity. The three other isolates scored negative (yellow colouration) for enzyme activity. Wells containing B6_2 gradually changed from yellow to orange indicating the presence of a carbapenemase. The color of the positive control assay changed from yellow to red during incubation at 37°C.

**Table 4.** Detection of carbapenemase activity by qualitative colorimetric ß-CARBA test.

	Color, T_0_	Color, T_30_	Carbapenemase activity
B6_2	yellow	orange	positive
B7_62	yellow	yellow	negative
B8_22	yellow	yellow	negative
B8_47	yellow	yellow	negative
*Pseudomonas aeruginosa* (CCUG 59347)	yellow	orange/red	positive
*Klebsiella pneumoniae* (CCUG45421)	yellow	yellow	negative

#### Further characterization of carbapenemase activity

The results of the KPC/MBL confirm kit (Rosco), B6_2 showed a small inhibition zone (12 mm) surrounding the meropenem tablet (MRP10). This zone was increased by 21 mm/diameter resulting in a 33 mm/diameter zone in the presence of the MBL inhibitor dipicolinic acid (MRPDP tablet). In the MIC Test Strip Synergy Applicator System, strain B6_2, was shown to be resistant to imipenem (MIC = 32), whereas imipenem combined with EDTA resulted in a sensitive phenotype (MIC = 1). The results of both tests are in accordance with MBL-activity. The positive and negative controls gave the expected result.

### Genome assemblies of the four *Pedobacter* spp. isolates

Whole genome sequencing of four isolates was performed using the minION sequencing platform by Oxford Nanopore Technologies (ONT). This provided long reads (7–9 KB) which were assembled into draft genomes of single contigs. The reads of Illumina HiSeq PE150 sequencing were assembled into draft genomes of various number of contigs for the four isolates (Table 5). The hybrid assemblies, merging the Illumina and minION reads into genome assemblies for all four isolates resulted in single contigs with a coverage of between 59–209 × (Table 5). No extrachromosomal elements were detected for either of the assemblies. The genome size of the hybrid assemblies compared to the draft genomes of minION and Illumina reads, were similar for all four isolates (Table 5). The genomes of B6_2 and B7_62 have similar genome sizes to *Pedobacter suwonensis* (NZ_FOJM00000000.1) and *Pedobacter terrae* (NZ_FNCH00000000.1), the two species to which they are both closely related (Fig. 1). The genome size of isolate B8_22 (4.3 MB) differed by more than 1.3 MB from the other genomes (Table 5). This is similar to the genome sizes of strains of the closely related species *Pedobacter antarcticus* (ATCC 51969, DSM 11 725, 4BY; genome sizes 4.4–4.5 MB). Isolate B8_47 and the closely related *Pedobacter cryoconitis* ASM159060v1 (Fig. 1) have a genome size of 5.9 MB.

**Table 5.** Genome assembly results.

		B6_2	B7_62	B8_22	B8_47
Number of contigs	minION	1	1	1	1
	Illumina	49	61	21	58
	Hybrid	1	1	1	1
Coverage/medium depth	minION	28.6 ×	29.5 ×	39.4 ×	28.1 ×
	Illumina	260 ×	292 ×	568 ×	270 ×
	Hybrid	59.1 ×	72.4 ×	209 ×	65.5 ×
Genome size (MB)	minION	5.7	5.6	4.3	5.9
	Illumina	5.7	5.5	4.2	5.8
	Hybrid	5.7	5.6	4.2	5.8
DNA G+C content (%)	minION	38.5	38.7	40.8	36.7
	Illumina	38.3	38.5	40.6	39.6
	Hybrid	38.3	38.4	40.6	39.6

### Phylogeny analysis of *Pedobacter* spp.

Figure 1 shows a phylogenetic tree of 32 *Pedobacter* strains. The four isolates analyzed in this study spread out on two different clades. Isolates B7_64 and B6_2 have a similar phylogeny and group together as nearest neighbors. Isolate B8_22 is closely related to *Pedobacter antarcticus*, while isolate B8_47 branches with *Pedobacter cryoconitis*. Fig. 1 suggests that there are three main clades of *Pedobacter* spp. Based on the different colony morphologies and colors of the isolates investigated in this study, a survey of the available literature on the appearance of *Pedobacter* spp. was performed. The results showed that colony coloration seems to follow the assignment of *Pedobacter* to the two larger clades. Clade 2 contains chiefly pink/red pigmented colonies, whilst Clade 3 contains chiefly white/yellow colonies. This was in accordance with the observed color of the four isolates analyzed in the present study.

### Resistome analysis

**Figure 2.**
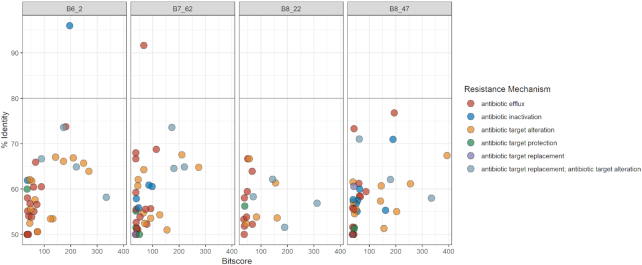
Bit score vs. identity distribution of resistance gene sequences identified in minION draft genomes by RGI in the CARD database according to resistance mechanism with a 50% cut-off on the identity score.

**Figure 3.**
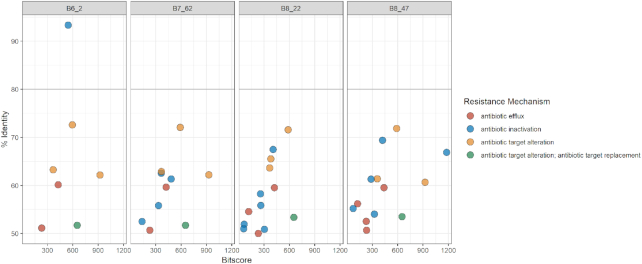
Bit score vs. identity distribution of resistance gene sequences identified in Illumina draft genomes by RGI in the CARD database according to resistance mechanism with a 50% cut-off on the identity score.

**Figure 4.**
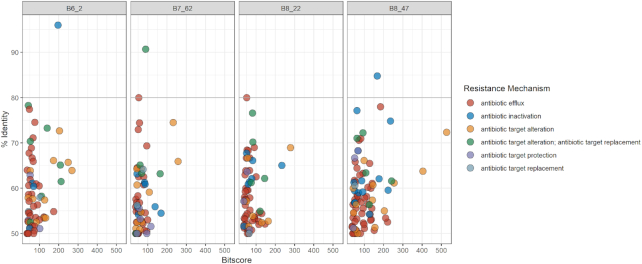
Bit score vs. identity distribution of resistance gene sequences identified in minION-Illumina-hybrid genomes by RGI in the CARD database according to resistance mechanism with a 50% cut-off on the identity score.

The resistance profiles obtained using RGI show that a number of antibiotic resistance ontologies (ARO) in the four draft genomes align fully or partially to homologues in the CARD database. As seen in Fig. 2, B6_2 and B7_62 were the only isolates where resistance gene sequences with a percentage identity above 80% were identified in the four minION draft genomes. For B6_2, one sequence showed a 96% similarity to *pedo-2*, a gene coding for a B3 metallo-ß-lactamase (MBL). The r*osA* gene, which is part of the RosAB potassium antiporter efflux system (Bengoechea and Skurnik 2000), was detected in B7_62 with a 91% identity score to the reference sequence in CARD. The resistance profiles of the four Illumina draft genomes (Fig. 3) gave fewer hits at the 50% identity cut-off when compared to minION and hybrid data. Moreover, only in isolate B6_2 was RGI able to detect a homologue in CARD with a percentage similarity above 80%. The putative *pedo-2* gene has a 96% similarity to the reference in CARD. Although less numerous, the AROs identified in the Illumina draft genomes had overall higher bitscores than those found in the minION assemblies. The RGI results of the hybrid genomes (Fig. 4) provided AROs of 80% identity scores or above for all four isolates. In addition to the *pedo-2* gene detected in B6_2, the *bcrA* gene coding for an ATP-binding cassette (ABC) antibiotic efflux pump (Podlesek *et al*. 1995) was identified in strain B6_72. Additionally, RGI highlighted a mutation in the *rpoB* gene (RNA polymerase ß-subunit) which has been associated with rifampicin resistance in the CARD database. In B8_22, *mexI* (coding for a component of the resistance-nodulation-cell division (RND) multidrug efflux pump family (Aendekerk *et al*. 2005)) was detected with an 80% similarity, *rphB* (rifampin phosphotransferase protein (Pawlowski *et al*. 2016)) was detected with 85% similarity score in B8_47. The *pedo-2* gene was the only resistance-associated gene detected in the same isolate in all three assembly approaches (minION, Illumina and hybrid). The *pedo-2* sequence from B6_2 Illumina sequence data was aligned with the reference sequence NG_04 9958.1 of Gudeta *et al*. (2016). The alignment shown in Supplementary Figure 1a, illustrates that the two sequences have a similarity of 85.77%. Furthermore, the translated protein sequence of the B6_2 PEDO-2 was aligned with a 100% similarity with the Subclass B3 metallo-beta-lactamase PEDO-2 protein (sequence ID: WP_0 638 6459.1) in BLASTP (Supplementary Figure 1b).

**Figure 5.**
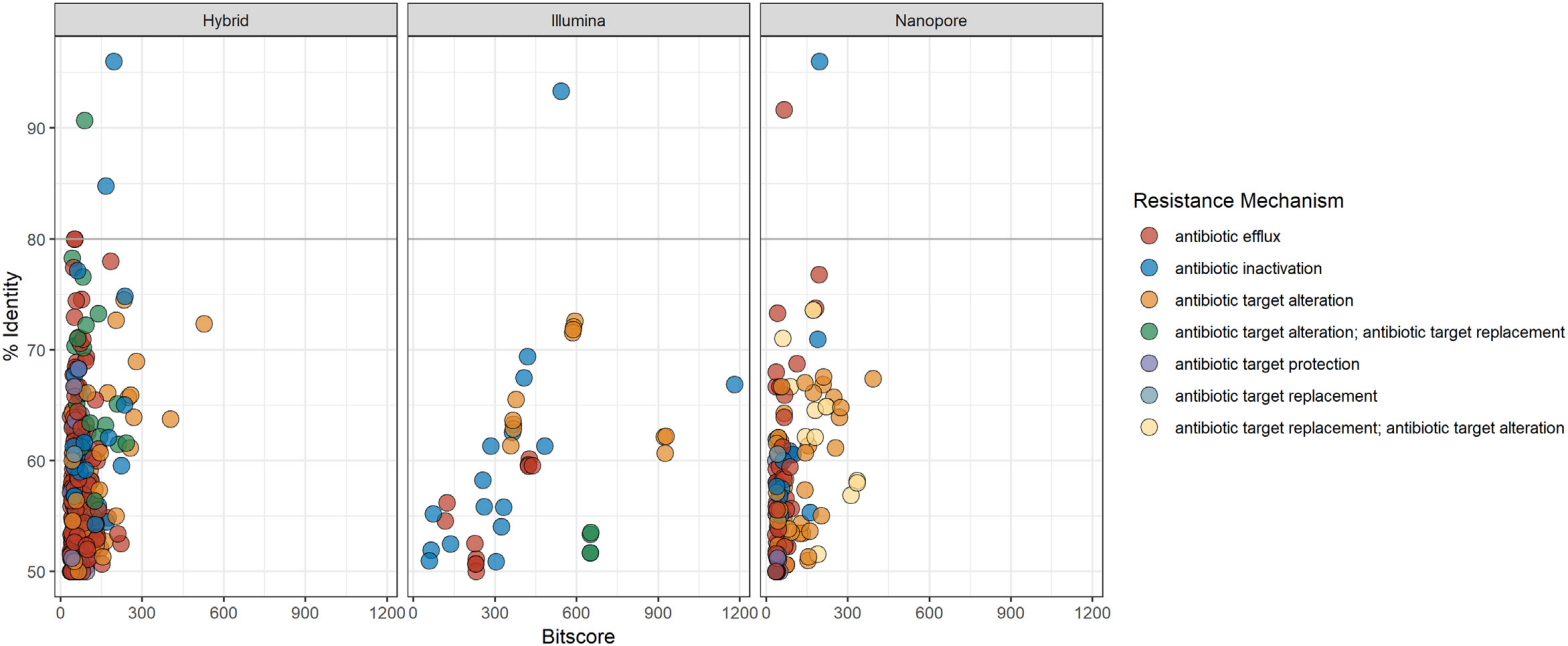
Bit score vs. identity distribution of resistance gene sequences identified in minION, Illumina and hybrid genomes draft genomes by RGI in the CARD database according to resistance mechanism with a 50% cut-off on the identity score. For the three panels, data from all four isolates are combined to illustrate the accuracy of the three assembly methods in resistance profile search.

Figure 5 highlights the major differences of the three assemblies combining information on all four isolates for each approach (minION, Illumina HiSeq PE150 and hybrid). For the eight genes with a percentage identity above 80%, half would confer resistance by antibiotic inactivation, three of these genes are related to efflux, while one is associated with antibiotic target alteration.

Figures 2–5 show that the most abundant mechanism of resistance identified for all four isolates when using a 50% identity cut off is antibiotic efflux. The identified AROs associated with antibiotic efflux belong to four of the five described multidrug efflux transporter systems, as shown in Supplementary Table 2. The results from RGI did not detect any genes belonging to the multidrug and toxic compound extrusion (MATE) family. The majority of the genes associated with antibiotic efflux identified in this study, belong to the resistance-nodulation-cell division (RND) family, a tripartite efflux system that spans the double membrane of Gram-negative bacteria. They have the ability to transport a variety of antibiotics out of the bacteria cell as a response to external stress factors (Dreier and Ruggerone 2015; Venter *et al*. 2015).

The online tool ICEfinder (Liu *et al*. 2018) did not detect any integrative or conjugative elements related to the mobilization of ARG in the flanking regions surrounding the putative resistance genes identified by RGI. The flanking sequences of the *pedo-2* gene detected in strain B6_2 was analyzed in more depth for mobile genetic elements. However, none were detected.

## DISCUSSION

The role of the environmental resistome in the evolution and spread of antibiotic resistance genes has received increasing attention over the last decades (Bengtsson-Palme, Kristiansson and Larsson 2018; Berendonk *et al*. 2015; Cantas *et al*. 2013; Viana *et al*. 2018b). However, owing to the lack of a good experimental framework, there are still challenges when working with environmental samples and bacteria in the context of resistance (Larsson *et al*. 2018). The use of whole genome sequencing (WGS) combined with phenotypic analyses performed in this study, has provided information on the patterns and mechanisms of resistance in four drinking water sludge *Pedobacter* isolates. In addition, this study has extended the phylogeny of what has been called an environmental superbug (Viana *et al*. 2018b).

All *Pedobacter* isolates investigated in this study showed phenotypic resistance to several clinically important antibiotics, and can be classified as multidrug resistant (MDR) bacteria (Magiorakos *et al*. 2012). Imipenem was one of three antibiotics tested that had a variable effect on the growth of the four isolates chosen for further study. B7_62 and B8_22 were both sensitive to imipenem, B8_47 showed an intermediate resistance, whilst isolate B6_2 showed complete resistance to this beta-lactam, perhaps indicating carbapenemase activity. These results were in line with the measured MIC values (Table 3) of the four isolates for imipenem. The MIC values determined for three additional carbapenems (doripenem, ertapenem and meropenem) showed that B6_2 in particular is generally carbapenem resistant. The presence and activity of a number of *Pedobacter* MBL have been demonstrated mainly after expression in *E. coli* (Gudeta *et al*. 2016b; Viana *et al*. 2018b) but also in supernatants of the *Pedobacter* of origin (Gudeta *et al*. 2016a). In the latter instance, activity was detected in supernatants of soil isolates of presumptive *P. roseus, P. borealis and P. kyungheenis*, and these were shown to harbor new MBL designated PEDO 1–3, respectively. The detection of a putative *pedo-2* MBL-gene in isolate B6_2 by RGI, might explain wholly or in part the observed carbapenem resistances in this isolate. A number of tests, including commercially available kits have been developed to assay for the activity of carbapenamase in general and MBL specifically. The latter mainly exploit the presence of metallic ions in the active site of MBLs which would be chelated by EDTA or dipicolinic acid (Alakomi *et al*. 2006; Chen *et al*. 2017).For strains B8_22 and B7_62, no evidence of carbapenemase activity was found, and this was in accord with antibiotic sensitivity assays and genome data. The resistance/intermediate susceptibility of strain B8_47 to several carbapenems was not related to carbapenamase activity. Furthermore, the genetic data did not find evidence of candidate carbapenem-inactivating genes. It is possible that the resistant/intermediate resistant phenotype could be attributed to other mechanisms of resistance, not as yet identified. In addition to the finding of a putative MBL gene, strain B6_2 was resistant to carbapenems and MBL activity was detected by two commercially available kits designed for this purpose. However, neither the CIM nor modified Hodge tests detected carbapenemase activity. These tests are based on indicator strain rescue, where ‘detection’ of MBL-activity depends on the MBL-producer inactivating sufficient amounts of carbapenem to have a measurable effect on the growth of the indicator strain. This would perhaps make these assays less sensitive than the other tests investigated, where MBL-activity in Pedobacter is assayed for directly. The putative *pedo-2* sequence detected in B6_2 was shown to be of high similarity to the blaB3PEDO subclass B3 MBL *pedo-2* (Supplementary figure 1) (Gudeta *et al*. 2016b). No complete or partial MBL sequences were detected in the other three isolates, which was also consistent with the phenotypic results (Table 2–4).

In the data generated by RGI against homologues in CARD, the most abundant potential mechanisms of resistance detected in the four genome assemblies were efflux pumps. The high number and diversity of efflux systems reported in this study, could explain several of the observed resistance patterns. Furthermore, they may aid the survival of *Pedobacter* in hostile environments such as drinking water sludge, with expected high concentrations of metals and other solutes used in drinking water clarification. The presence of heavy metals in the environment can lead to an increased selection pressure for the development of antibiotic resistance even in the absence of antibiotics, as several of the regulators of efflux systems are induced by the presence of metals (Blair *et al*. 2015; Seiler and Berendonk 2012; Sun *et al*. 2014; Yu *et al*. 2017). It is possible that the high concentrations of for example metals and polymers arising during sludge production, could create a toxic environment forcing an upregulation of efflux systems (Nies 2003; Pal *et al*. 2017). This intrinsic mechanism of resistance has also been found to be mobilizable on plasmids (Martinez *et al*. 2009) and thereby potentially transferable to other bacteria including pathogens. However, we did not find any mobile genetic elements surrounding the annotated efflux systems, nor were there any extrachromosomal elements detected in the four isolates. Efflux transporter systems function often in tripartite structures (RND and MFS) requiring multiple domains for functionality (Jones *et al*. 2015; Piddock 2006; Sun *et al*. 2014). In the present study, several of the genes identified that were related to efflux could be placed in groups of known systems. However, some of the components were not present in either of the datasets. Sequence similarities within transporter families, such as *ade-, acr-* and *mex-* systems of the RND-family has been found, and may functionally substitute for one another in certain species (Piddock 2006; Venter *et al*. 2015). Whether this is the case for *Pedobacter* is not at present known. A functional study on efflux transporter systems in the genus would be useful. In addition, studies aimed at investigating if there are connections between antibiotic resistance and possible efflux-based adaptations of this genus to hostile environments, are worthy of further attention.

The CARD results presented, show large differences when using respectively 80% and 50% identity scores as cut-offs (Figs 2–5). It has previously been shown that using a threshold of 80% for identification of resistance genes in environmental bacteria, may create a large number of false negatives (Arango-Argoty *et al*. 2018). The algorithms used to create CARD and similar databases are biased towards clinical isolates, and thus important data may be lost or not identified when using them to examine environmental isolates. Illumina data is more accurate than that generated by minION sequencing (Pfeiffer *et al*. 2018), and will produce higher similarity scores to the reference sequences in CARD. For instance, in the B7_62 minION dataset, *rosA* was shown to have a percentage similarity above 90% to the reference gene in CARD. In the hybrid dataset, the same hit had a 69% similarity. However, the trends of the datasets provided by the three assembly methods are similar (Fig. 5). By combining data from the two sequencing platforms, the genomes produced provide better evidence of the hits detected by Illumina or minION sequencing alone.

The phylogenetic analysis of 32 *Pedobacter* genomes performed in this study, presents new insights into the diversity of this genus (Fig. 1). The four isolates investigated in depth, are clearly separated on two different clades. It is possible that B6_2 and B7_62, which branch together on Clade 2, represent a new species of *Pedobacter*. These isolates also have similar genome sizes (Table 5). However, in line with the standard approach to describing new species, extended phenotypic tests, including substrate utilization and a DNA: DNA hybridization assay need to be performed. It is also noteworthy that B8_22 has a significantly smaller genome size than the other three sequenced isolates (Table 5). The related species *Pedobacter antarcticus* (Farfan, Montes and Marques 2014) has approximately the same genome size as B8_22. Furthermore, two other species isolated from cold habitats, *Pedobacter arcticus* (Yin *et al*. 2012) and *Pedobacter himalayensis* (Shivaji *et al*. 2005), also have small genomes, 3.9 MB and 4.6 MB, respectively. The advantages of the small genome size of these strains and the connection, if any, to an adaptation to colder climates would be interesting to investigate in more depth. We note also that pigmentation of species of *Pedobacter*, divides itself strongly along the lines of the proposed clades (Fig. 1). This observation does not seem to have been made previously, and it will be interesting to see if changes to the genus taxonomy might be suggested in future studies.

To summarize, twenty-two new sludge isolates of the genus *Pedobacter* were found to be resistant towards several clinically important antibiotics. Some dedicated antibiotic resistance genes were demonstrated. However, it is possible that the MDR phenotype may in part be a consequence of more general survival mechanisms (e.g. efflux pumps) developed in response to other selective pressures, such as the hostile conditions represented by drinking water sludge. Metallo-beta-lactamase activity conferring or contributing resistance to carbapenems was demonstrated in one isolate. This is most likely due to the presence of *pedo-2*, a gene of the B3 PEDO beta-lactamase subclass. The present study also extends the phylogeny of the genus *Pedobacter*.

## Supplementary Material

fiaa088_Supplemental_Figures
