# Supplementary figures and images for: Whole genome sequencing and antibiotic diffusion assays, provide new insight on drug resistance in the genus Pedobacter
Source: FEMS Microbiol Ecol. 2020 May 9;96(6):fiaa088. doi: 10.1093/femsec/fiaa088 (PMC7254926; doi:10.1093/femsec/fiaa088)

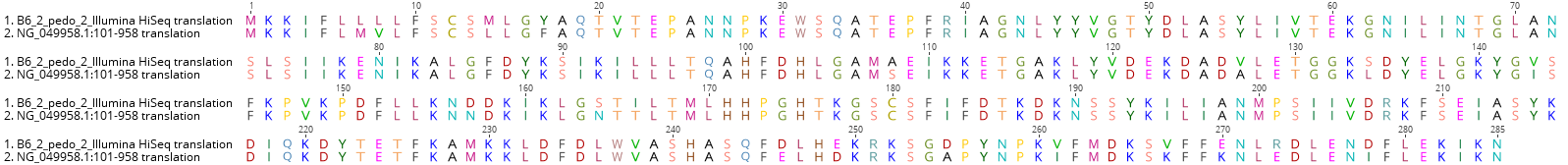

Supplement: fiaa088_Supplemental_Figures [file fiaa088_supplemental_figures.zip › Sup1b_translated.tif]
